# Efficient targeted recombination with CRISPR/Cas9 in hybrids of *Caenorhabditis* nematodes with suppressed recombination

**DOI:** 10.1186/s12915-023-01704-0

**Published:** 2023-09-29

**Authors:** Dongying Xie, Bida Gu, Yiqing Liu, Pohao Ye, Yiming Ma, Tongshu Wen, Xiaoyuan Song, Zhongying Zhao

**Affiliations:** 1 Department of Biology, Hong Kong Baptist University, Kowloon, Hong Kong SAR China; 2 Department of Quantitative and Computational Biology, University of Southern California, Los Angeles, USA; 3 MOE Key Laboratory of Cellular Dynamics, CAS Key Laboratory of Brain Function and Disease, School of Life Sciences, Division of Life Sciences and Medicine, Hefei National Research Center for Physical Sciences at the Microscale, University of Science and Technology of China, Hefei, 230026 Anhui China

**Keywords:** Targeted recombination, CRISPR/Cas9, *C. briggsae*, *C. nigoni*, Hybrid, Genetic mapping

## Abstract

**Background:**

Homology-based recombination (HR) is the cornerstone of genetic mapping. However, a lack of sufficient sequence homology or the presence of a genomic rearrangement prevents HR through crossing, which inhibits genetic mapping in relevant genomic regions. This is particularly true in species hybrids whose genomic sequences are highly divergent along with various genome arrangements, making the mapping of genetic loci, such as hybrid incompatibility (HI) loci, through crossing impractical. We previously mapped tens of HI loci between two nematodes, *Caenorhabditis briggsae* and *C. nigoni*, through the repeated backcrossing of GFP-linked *C. briggsae* fragments into *C. nigoni*. However, the median introgression size was over 7 Mb, indicating apparent HR suppression and preventing the subsequent cloning of the causative gene underlying a given HI phenotype. Therefore, a robust method that permits recombination independent of sequence homology is desperately desired.

**Results:**

Here, we report a method of highly efficient targeted recombination (TR) induced by CRISPR/Cas9 with dual guide RNAs (gRNAs), which circumvents the HR suppression in hybrids between the two species. We demonstrated that a single gRNA was able to induce efficient TR between highly homologous sequences only in the F1 hybrids but not in the hybrids that carry a GFP-linked *C. briggsae* fragment in an otherwise *C. nigoni* background. We achieved highly efficient TR, regardless of sequence homology or genetic background, when dual gRNAs were used that each specifically targeted one parental chromosome. We further showed that dual gRNAs were able to induce efficient TR within genomic regions that had undergone inversion, in which HR-based recombination was expected to be suppressed, supporting the idea that dual-gRNA-induced TR can be achieved through nonhomology-based end joining between two parental chromosomes.

**Conclusions:**

Recombination suppression can be circumvented through CRISPR/Cas9 with dual gRNAs, regardless of sequence homology or the genetic background of the species hybrid. This method is expected to be applicable to other situations in which recombination is suppressed in interspecies or intrapopulation hybrids.

**Supplementary Information:**

The online version contains supplementary material available at 10.1186/s12915-023-01704-0.

## Background

DNA double-strand breaks (DSBs) caused by endogenous or exogenous agents usually trigger DNA repair through homology-based recombination (HR) [1]. The crossover between homologous chromosomes teases apart genetic linkages, resulting in new combinations of alleles and the diversification of populational genetic architectures, which lay the foundations for genetic and phenotypic novelty [2]. Linkage analyses have revealed that recombination frequency varies substantially among species [3–5] or across various parts of a chromosome [6]. Efficient HR is essential for the genetic and molecular characterization of gene functions, especially for the fine mapping of a genetic locus.

**Fig. 1:**
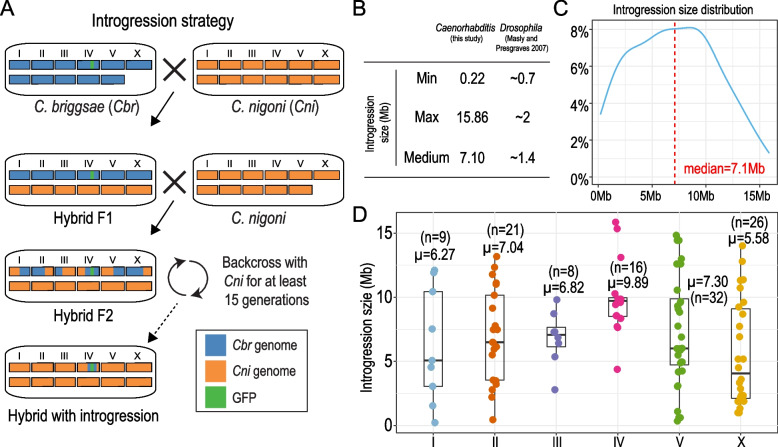
Recombination supression in the hybrids between *C. briggsae* and *C. nigoni*. **A** Schematic diagram showing the crossing strategies to generate introgression from the GFP-labeled *C. briggsae* genome (*Cbr*, blue bars) to the *C. nigoni* genome (*Cni*, orange bars) as a marker. Chromosome numbers are indicated. **B** Comparison of introgression fragment sizes between *Caenorhabditis* (this study) and *Drosophila* hybrids [10]. **C** Density plot showing the distribution of introgression size of 112 independently generated *C. nigoni* strains that each carries a *C. briggsae* introgression fragment. The median size of the introgression fragments is indicated by a red dashed line. **D** Box plots showing the individual introgression sizes of the 112 lines across chromosomes (differentially color-coded). The mean of introgression size for each chromosome is indicated on the top. Note that introgression fragments from the *C. briggsae* chromosome IV demonstrate the largest mean and median size (*p* < 0.05, Wilcoxon rank sum test with multiple testing correction using FDR method)

Unfortunately, in addition to crossover interference and an uneven distribution and frequency of crossover events within chromosomes, HR is also frequently suppressed by an elevated degree of sequence divergence or the presence of genomic rearrangements [7]. This is especially the case in species hybrids in which sequence divergence or the occurrence of genomic rearrangement is significantly higher than that between populations of the same species. For example, to empower nematodes *Caenorhabditis briggsae* and *C. nigoni* as models for speciation study, we previously created a genome-wide landscape of hybrid incompatibility (HI) by repeated backcrossing of individual GFP-labeled *C. briggsae* genomic fragment into *C. nigoni* (Fig. 1A) [8]. However, our attempt to determine the molecular identity of individual HI loci was unsuccessful because of the apparent lack of spontaneous recombination between homologous chromosome arms in the hybrids of the two species [8]. Genome sequencing revealed that pervasive genome rearrangements and an unusually high degree of sequence divergence between the two nematodes were plausible explanations for the reduced recombination efficiency [9].

To circumvent recombination suppression, multiple attempts have been made to achieve targeted recombination (TR). For example, site-specific recombinase systems such as Cre/*lox* and FLP/FRT have long been adopted for efficient TR [11, 12]. However, the prerequisite for the integration of recognition sequences at specific sites restricts its application. Other studies have demonstrated reasonable efficiency when combining the recombination-initiation protein Spo11 with various site-specific DNA-binding modules, including Gal4, zinc fingers, and transcription activator-like effector [13, 14]. As recombination is usually invoked upon DSBs, the emergence of the CRISPR/Cas systems opened new avenues for TR. Given that CRISPR/Cas-mediated gene knock-in relies on HR between the targeted genome and donor sequences, it was soon adopted for TR because it facilitates the easy selection of recombination sites. To date, TRs have been reported during both mitotic [15–17] and meiotic stages [18] across various taxa in studies using Cas9 or Cas12 [19] and were achieved mostly through HR by a single DSB on one of the homologous chromosomes within a species. However, it is not clear whether the system can be used for circumventing suppressed recombination in species hybrids, in which the level of sequence divergence and genome rearrangement can be significantly higher. Unfortunately, existing cloned interspecies HI loci are mostly located within genomic regions with fast rates of sequence divergence and genome rearrangement [20], making the mapping of HI loci difficult through spontaneous HR. Therefore, an efficient TR method is urgently needed to circumvent recombination suppression, especially for the molecular cloning of interspecies HI loci. In this study, we demonstrated that efficient TR can be achieved through CRISPR/Cas9 with dual gRNAs in species hybrids, independently of sequence homology and genetic background.

## Results

### An unusually large size of introgression fragments indicates that spontaneous HR is suppressed in the hybrids between *C. briggsae* and *C. nigoni*

To systemically identify HI loci and their causative genes in the hybrids of *C. briggsae* and *C. nigoni*, we previously generated 112 independent hybrid lines, each carrying an introgression derived from a GFP-marker-linked *C. briggsae* genomic fragment on an otherwise *C. nigoni* background via repeated backcrossing [8, 21] (Fig. 1A). Specifically, we first generated 96 independent *C. briggsae* transgenic strains, each expressing a chromosomally integrated GFP marker. We next backcrossed each of the 96 GFP markers into *C. nigoni* for at least 15 generations, as previously described [10] (Fig. 1A). However, the introgression fragment size in the hybrid of *C. briggsae* and *C. nigoni* was approximately five times larger than that in similar *Drosophila* hybrids [10]. For example, the median and maximum introgression sizes in the *Caenorhabditis* hybrids were approximately 7.10 Mb and 15.86 Mb compared to approximately 1.4 Mb and 2 Mb in the *Drosophila* hybrids, respectively, although the minimum sizes were comparable (Fig. 1B, C) [10]. Most of the introgression sizes fell within a 5 Mb to 10 Mb range, even after backcrossing over 15 generations. Although both large and small introgression sizes were seen on all chromosomes, a significantly larger mean (9.89 Mb) or median (9.725 Mb) of the introgression sizes was observed on chromosome IV than other chromosomes (*p* < 0.05, Wilcoxon rank sum test with multiple testing correction using FDR). The results indicate that HR was suppressed between the homologous chromosomes of the two species in the hybrid strains, with chromosome IV showing the highest level of suppression (Fig. 1D and Additional File 2: Table S1).

### Targeted recombination can be achieved in regions with a relatively high degree of sequence homology in hybrid F1s but not introgression strains by single gRNA

**Fig. 2:**
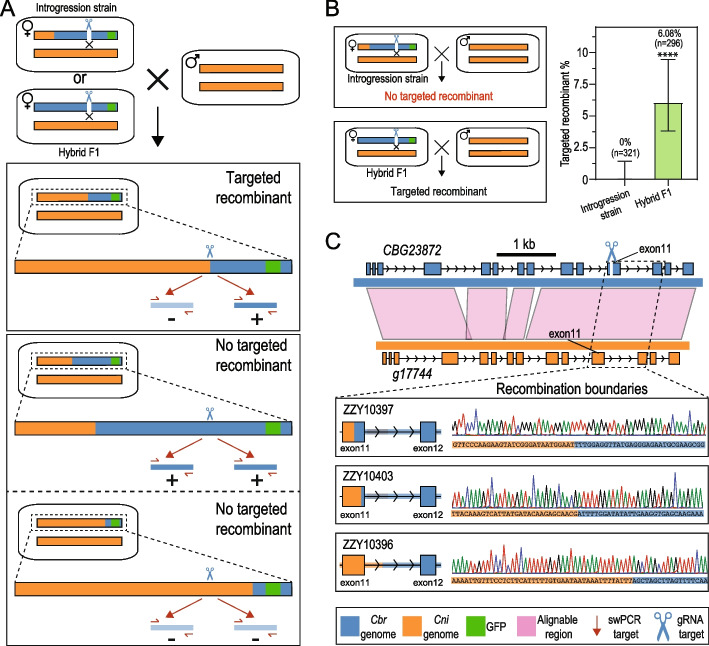
*C. briggsae-*specific gRNA induces targeted recombination in the hybrid F1 progeny between *C. briggsae* and *C. nigoni* but not in the introgression strains. **A** Schematic diagram showing the generation and validation of targeted recombination on chromosome IV. Specifically, a double strand break is induced by the injection of Cas9 and a *C. briggsae*-specific gRNA into the females of an introgression strain (ZZY10458) or of the F1 hybrids between ZZY0734 male and *C. nigoni* female. The right arm of the *C. briggsae* chromosome IV is marked with a GFP insertion. The F1 hybrid or the introgression females are backcrossed to *C. nigoni* wild isolate males. The crossing progeny are screened for the presence of targeted recombinant by single-worm PCR (swPCR) amplification of the *C. briggsae*-specific genomic fragments flanking the expected target site. The absence ( −) and presence ( +) of a PCR product on the left and right side of the target site indicate a successful targeted recombination. Simultaneous presence or absence of PCR product(s) on both sides of the target site indicates no targeted recombination. For simplicity, only the chromosome IV is shown. **B** Targeted recombinant is absent in the crossing progeny between the introgression strain and *C. nigoni* (*top left*) but is present in crossing progeny between the hybrid F1 and *C. nigoni* (*bottom left*) using the *C. briggsae*-specific gRNA targeting gene *CBG23872*. Shown on the right is the bar plot of targeted recombinant frequency with the total number of screened worms (*n*) indicated. Error bar represents 95% confidence interval calculated with the Agresti-Coull method. (****) *p* < 0.0001 (chi-square test). **C** Validation of the targeted recombinants in the hybrid F1 progeny (**B**) through sequencing the recombination boundaries. Top: the sequence alignment (pink block) flanking the gRNA target site. Bottom: sequencing results for the boundaries of three independent targeted recombinants

Given that a DSB on one of the homologous chromosomes triggers HR, we reasoned that a DSB artificially induced through CRISPR/Cas9 could facilitate TR in the hybrids of *C. briggsae* and *C. nigoni*. To test this, we focused on the genes located on the chromosome IV, the HR of which showed a relatively higher level of recombination suppression than those of the remaining chromosomes (Fig. 1D). We injected ribonucleoproteins (RNPs) consisting of Cas9 and a *C. briggsae*-specific gRNA into the female germline of a hybrid F1 or introgression strain in three replicates. Specifically, the hybrid F1 animals were produced by crossing *C. briggsae* transgenic males carrying a GFP marker on the chromosome IV (ZZY0734) with *C. nigoni* wild isolate female (JU1421). The transgenic *C. briggsae* animals were then backcrossed with *C. nigoni* for at least 15 generations to generate an introgression line expressing GFP, i.e., ZZY10458 (Fig. 1A). We selected a *C. briggsae-*specific gRNA that targeted the gene *CBG23872*, which is syntenic to its *C. nigoni* ortholog *g17744*. The two genes showed over 90% sequence similarity within both coding and intronic sequences (Fig. 2C). The gRNA specifically targeted the eleventh exon of *CBG23872* but not *g17744* based on PAM polymorphism as the others did [15, 16, 22, 23] (Fig. 2C), so that DSB was expected to occur only on the *C. briggsae* chromosome.

Our initial purpose was to reduce the interval of the introgression fragment carrying the gene linked to GFP. Therefore, we first injected the RNPs into the female gonads of the introgression line, ZZY10458. The injected animals were then mated with *C. briggsae* males. We screened for the presence of recombinants through single-worm PCRs (swPCRs) of individual F1 females by using two pairs of *C. briggsae-*specific primers flanking the expected target site (Fig. 2A). The primers were located within a 2-kb distance from the gRNA target site because HR usually occurs within a few kilobase pairs from the DSBs [24]. The presence of a PCR product at one side but not the other side of the target site indicates a successful TR, whereas the simultaneous presence or absence of a PCR product indicates there is no TR, at least within the screened genomic interval (Fig. 2A). Unfortunately, we did not achieve any TR after screening 321 animals resulting from three sessions of injection (Fig. 2B), suggesting that CRISPR/Cas9-triggered DSB is still not a feasible way to induce HR in the introgression strain.

We previously showed that recombination between *C. briggsae* and *C. nigoni* homologous chromosomes mostly took place at the F1 or F2 generation [9]. Recombination was rarely observed after F2, suggesting that *C. nigoni* carrying an introgression somehow established a mechanism to prevent HR between syntenic regions after purging *C. briggsae* genomic fragments unlinked to the introgression fragment. This mechanism may not have been established in the F1 hybrids. Consistent with this, TR was successfully achieved in the hybrid F1s of another nematode intraspecies [22]. To test this, we injected the same RNPs as those used in the introgression strains into the gonads of the F1 hybrids (Fig. 1B). As expected, we obtained 18 targeted recombinants out of 296 worms genotyped (6.08%) using swPCR in three sessions of injection (Fig. 2B, Additional File 2: Table S2), which is significantly higher than the targeted recombinant frequency in the introgression strain (*p* < 0.0001, chi-square test). We randomly picked three targeted recombinants (ZZY10396, ZZY10397, and ZZY10403) for Sanger sequencing to examine their recombination boundaries (Fig. 1C). Indeed, all the TR events took place just a few hundred base pairs downstream of the gRNA target site, suggesting that the same DSB can induce multiple different crossover events nearby (Fig. 1C). Notably, in all three sequenced recombinants, and probably all targeted recombinants, crossover events took place downstream of the expected *C. briggsae* DSB site, leading to the loss of the gRNA target sites in the *C. briggsae* genome in the hybrid (Fig. 2C). This is expected because if a crossover took place upstream of the target site, we would expect an intact gRNA target site in the hybrid, which would be targeted repeatedly by the same gRNA in the hybrid to produce a DSB. Taken together, the results demonstrated that efficient HR-based TR can be achieved within syntenic regions in the hybrid F1 but not in the *C. nigoni* strain carrying an introgression of *C. briggsae* where HR was somehow suppressed.

### Dual gRNAs each targeting *C. briggsae* and *C. nigoni* chromosomes respectively significantly improve efficiency of TR in hybrid F1s

**Fig. 3:**
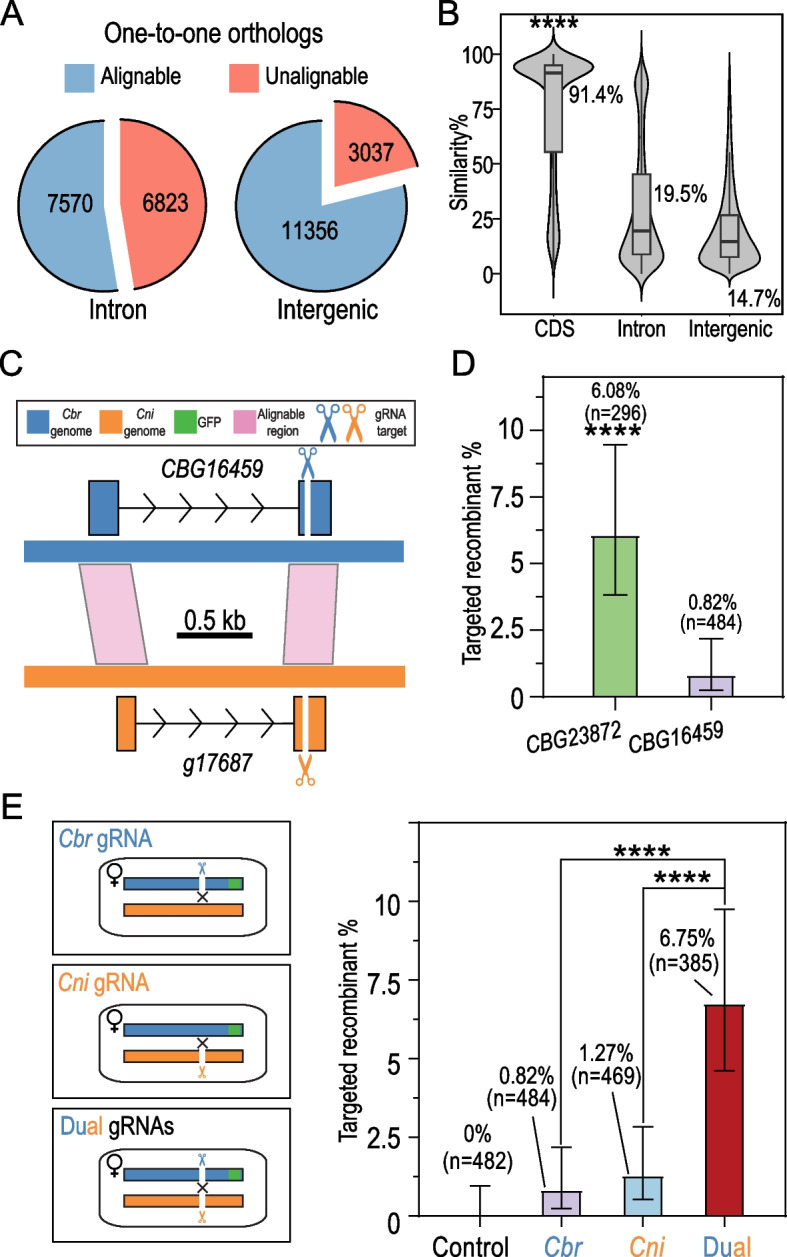
Injection of dual gRNAs individually targeting the *C. briggsae* and the *C. nigoni* genome significantly increases the efficiency of targeted recombination. **A** Pie charts showing the ratio between alignable (*blue*) and unalignable (*brown*) sequences for the introns and intergenic regions of one-to-one ortholog pairs between *C. briggsae* and *C. nigoni*. **B** Violin plots with overlaid boxplots showing the similarity of alignable coding sequences (CDS), introns, or intergenic sequences of one-to-one ortholog pairs. (****) *p* < 0.0001 (Wilcoxon ranked sum test with multiple testing correction using FDR method). **C** Shown is a recombination resistant region with a relatively low sequence homology and two gRNAs each targeting *C. briggsae* (*CBG16459*) and *C. nigoni* (*g17687*), respectively. **D** Bar plots showing the comparison of targeted recombinant frequency between the regions with a relatively high (Fig. 2D) and low sequence homology (**C**) using *C. briggsae-*specific gRNAs. (****) *p* < 0.0001 (chi-square test). Number of screened crossing progeny (*n*) is indicated in parenthesis. **E** Comparison of targeted recombinant frequency using a single or dual gRNA(s). Left: schematic diagrams of double-stranded DNA breaks induced by a single or dual species-specific gRNA(s). Right: bar plots showing the comparison of recombinant frequency between the control (no gRNA), and the treatment with a single or dual species-specific gRNA(s). Note that the animals treated with dual gRNAs display a significantly higher targeted recombinant frequency. (****) *p* < 0.0001 (Fisher’s exact test with multiple testing correction using FDR method)

Efficient HR depends on extensive sequence similarities between homologous chromosomes. *C. briggsae* and *C. nigoni* are closely related species. However, in addition to the numerous genomic rearrangements, their genome sizes and sequences are substantially divergent from each other, which at least partially explains the widespread suppression of recombination in their hybrids. To gain a detailed view of sequence similarity between the two genomes, we compared the sequences of all the one-to-one orthologs between *C. briggsae* and *C. nigoni* by contrasting the alignment-length-weighted similarities between each ortholog pair (see the “Methods” section). The results showed that nearly half of the introns (6823 or 47.4%) and approximately 21.1% of the intergenic regions of the ortholog pairs were unalignable (Fig. 3A). Although the similarity of the orthologous cDNA sequences was quite high (approximately 91.4%), that of the alignable orthologous introns and intergenic regions dropped to approximately 19.5% and 14.7%, respectively, which are significantly lower than that of the CDSs (*p* < 0.0001, Wilcoxon ranked sum tests with multiple testing correction using FDR) (Fig. 3B). The results showed that most of the *C. briggsae* genome may not be amenable to spontaneous HR, and the efficient TR we achieved in the region with relatively high sequence similarity (Fig. 2C) may not be applicable to regions with relatively low sequence similarity. To test this, we examined another single gRNA that targets the second exon of the *C. briggsae* gene *CBG16459*, which showed only approximately 80% similarity to its *C. nigoni* ortholog *g17687*. In addition, most of the introns and sequences flanking the two orthologs were unalignable with each other (Fig. 3C). Our previous data demonstrated that there was no spontaneous recombination between the two syntenic regions involving the orthologs upon crossing over 15 generations [10]. Indeed, we observed a dramatically reduced efficiency of TR between the two genes, i.e., 4 out 484 (0.82%) when compared with that between *CBG23872* and *g17744* (6.08%) (*p* < 0.0001, chi-square test; Fig. 3D and Additional File 2: Table S2). The reduced TR efficiency was unlikely to be attributed to the differences in gRNA sequences because the gRNA targeting *CBG16459* even demonstrated a higher specificity score and predicted efficiency than that targeting *CBG23872* (Additional File 2: Table S3). To examine whether the low efficiency was associated with the *C. briggsae*-specific gRNA, we injected another *C. nigoni*-specific gRNA targeting the syntenic exon. The results only showed a modest improvement in recombination efficiency, i.e., targeted recombinant frequency of approximate 1.27% versus 0.82% for *C. briggsae-*specific gRNA (*p* = 0.5416, Fisher’s exact test, Fig. 3E). Given that the spontaneous recombination rate also exhibits regional variations across the chromosome, with lower and higher rates observed in the center and arms of the chromosomes, respectively, it is possible that the increased TR efficiency could be a product of genomic position. To test the effect of chromosomal positions on the TR efficiency, we selected another four *C. briggsae* genes that are located in the middle of the same chromosome (IV) (Additional File 1: Fig. S1). These four genes were selected so that their sequence homology is higher than that of *CBG16459*, which is located on the right arm of the chromosome IV. Although that the recombinant rate on the chromosome arm is expected to be higher than that in the middle of the chromosome, and the predicted efficiency scores of gRNAs for the four genes are comparable with or lower than that of *CBG16459* (Additional File 2: Table S3), all four genes demonstrated a significantly higher TR efficiency that that *of CBG16459* (Additional File 1: Fig. S1). Taken together, our results suggest that the efficiency of TR using a single species-specific gRNA can vary considerably depending on the target site. The elevated level of sequence homology may contribute to the observed increase in the frequency of targeted recombination.

If a recombination event is triggered by a single gRNA, it is expected to take place through crossover that is dependent on sequence homology, which is much less efficient than the recombination through non-homology-based end joining (NHEJ). We reasoned that the creation of dual DSBs, with one in each of the *C. briggsae* and *C. nigoni* genomes, would be likely to improve the recombination frequency through NHEJ (Fig. 3E). As expected, when we simultaneously injected both *C. briggsae-* and *C. nigoni-*specific gRNAs targeting *CBG16459* and *g17687* respectively, into the F1 hybrids, a significantly higher efficiency of TR was achieved, i.e., 26 out of 385 (6.75%), when compared with that of TR using single species-specific gRNA (*p* < 0.0001, Fisher’s exact tests with multiple testing correction using FDR; Fig. 3E). This result demonstrated that dual gRNAs targeting both homologous chromosomes significantly improved the efficiency of TR in the F1 hybrids, regardless of sequence homology.

### Dual gRNAs can mediate TR within genomic regions that have undergone an inversion via NHEJ

**Fig. 4:**
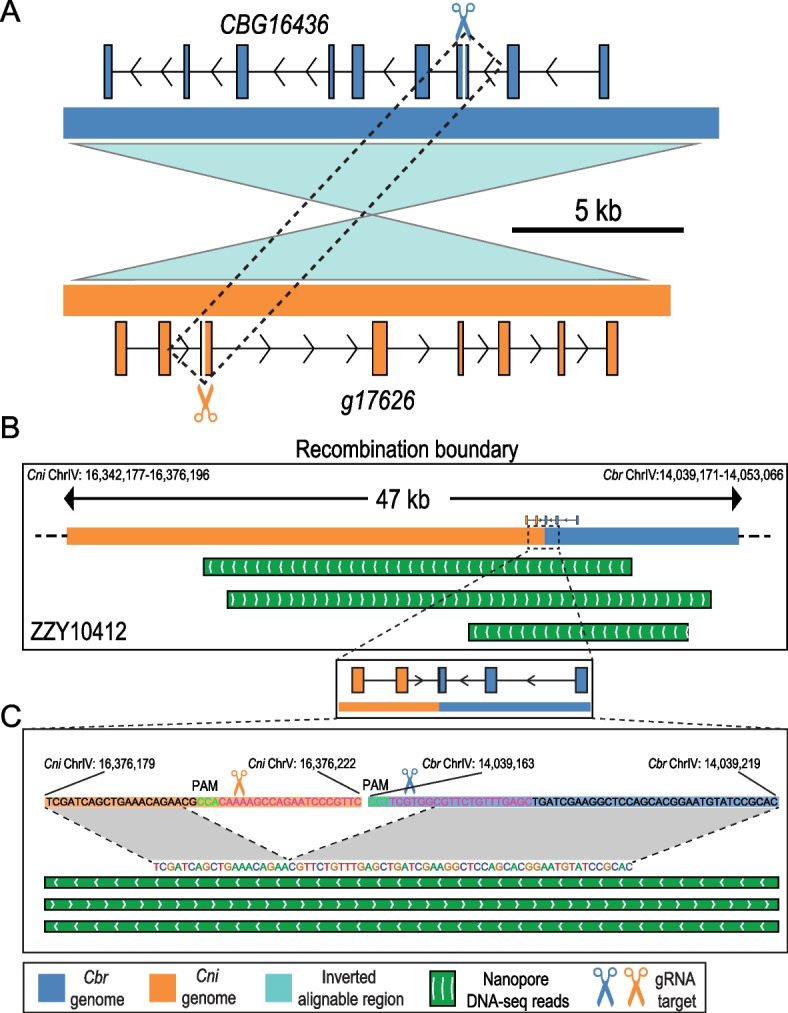
Dual gRNAs induce targeted recombination within a region that undergoes an inversion. **A** Schematic diagram showing two gRNAs each targeting the *C. briggsae* (*top*) and the *C. nigoni* genomes (*bottom*), respectively. Note that the syntenic genomic sequences between the two species are alignable but undergo an inversion, which prevents the recovery of any viable progeny resulting from homology-based recombination. **B** Confirmation of a targeted recombinant within the inverted region induced by the dual gRNAs through Nanopore DNA sequencing. Shown are sequencing reads and the gene models that span the recombination boundary. **C** A magnified view of the recombination boundary. The gRNA sequences and PAM sequences are highlighted in purple and green, respectively

The elevated TR efficiency between *CBG16459* and *g17687* mediated by dual gRNAs could be due to either an increased rate of HR or the creation of dual DSBs followed by DNA repair by NHEJ. To further confirm whether a TR can be triggered by the creation of dual DSBs followed by DNA repair through NHEJ, we focused on the *C. briggsae* gene *CBG16436* and its *C. nigoni* ortholog *g17626*, between which an inversion took place (Fig. 4A), and therefore, any hybrids resulting from the HR within the region were not expected to be viable due to aneuploidy. We injected a *C. briggsae-*specific gRNA targeting the third exon of *CBG16436* and a *C. nigoni*-specific gRNA targeting the same exon of *g17626* along with Cas9 proteins into the gonads of hybrid F1 females. We achieved a total of 10 targeted recombinants out of 98 worms screened, 6 of which were located within the gene bodies of *CBG16436* and *g17626*.

To determine the recombination boundaries, we randomly selected one recombinant and performed Oxford Nanopore DNA sequencing. Multiple boundary-spanning reads supported the conclusion that recombination occurred between *CBG16436* and *g17626* (Fig. 4B–C and Additional File 1: Fig. S4). The consensus of boundary-spanning reads revealed the deletion of 8–9 nucleotides and 4–5 nucleotides in the *C. nigoni* and *C. briggsae* chromosomes, respectively (Fig. 4C). The results indicated that recombination between *CBG16436* and *g17626* was achieved through NHEJ instead of HR due to the presence of the inversion.

Interestingly, in addition to the recombination that occurred at the above regions, we also identified another strain, ZZY10413, which showed a recombination between the *C. briggsae* gene *CBG16437* (approximately only 4 kb downstream of the *C. briggsae* gRNA target site) and its *C. nigoni* ortholog *g17641* (approximately 100 kb downstream of *g17626*) by Nanopore sequencing of the boundary (Additional Files 1: Fig. S2A and B, Fig. S4). Such recombination was likely to be achieved by *C. briggsae* gRNA-triggered HR, which resulted in the deletion of approximately 100 kb for the *C. nigoni* genome in the recombinants. A similar large deletion was observed when we examined the feasibility of NHEJ-based recombination at another HR-suppressed region between *C. briggsae* gene *CBG16450* and its *C. nigoni* ortholog *g17645*, which also underwent an inversion (Additional File 1: Fig. S3A). Besides the total of seven targeted recombinants, we obtained another strain, ZZY10422*,* whose recombination boundary was located approximately 90 kb downstream of the *C. briggsae* gRNA target site. The recombination boundary was further confirmed by Nanopore sequencing (Additional Files 1: Fig. S3B and Fig. S4). This crossover was unlikely to have been caused by HR because no sequence similarity was observed between the sequences flanking the recombined region. These results show that dual gRNA-triggered TR may produce unintended crossover events distant from the target region.

### Dual gRNAs mediate targeted recombination in the introgression strains with an efficiency comparable to that in the F1 hybrid of *C. briggsae* and *C. nigoni*

**Fig. 5:**
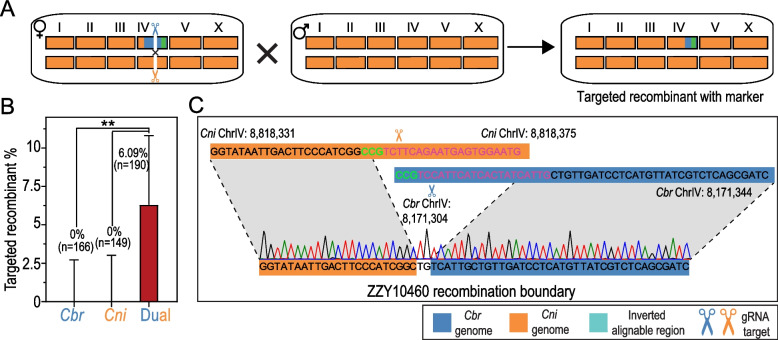
Dual gRNAs induce targeted recombination in the introgression strain. **A** Schematic diagram of species-specific dual gRNAs that induce DSBs in an introgression strain. For simplicity, recombinant carrying *C. briggsae* fragments without the GFP marker is not shown. *C. briggsae* and *C. nigoni* sequences are differentially color coded as in Fig. 1. **B** Comparison of targeted recombinant frequency in the progeny of introgression strain induced by a single species-specific gRNA and dual gRNAs. The number of total screened worms (*n*) are also indicated. Error bar represents 95% confidence interval calculated as in Fig. 2B. (∗ ∗) *p* < 0.01 (Fisher’s exact test with multiple testing correction using FDR method). **C** Confirmation of the targeted recombinant induced by the dual gRNAs through Sanger DNA sequencing. The gRNA sequences and PAM sequences are highlighted in purple and green, respectively

Given that TR can be achieved through two gRNAs independent of sequence homology, we reasoned that dual gRNAs might also be able to induce TR in the introgression strains in which a single gRNA failed to induce TR, even when there was high sequence homology. To this end, we injected dual gRNAs targeting the *C. briggsae* gene *CBG05992* and its *C. nigoni* ortholog *g15658* into the same introgression strain (ZZY10458), as shown in Fig. 2B (Fig. 5A). As a control, we first injected each gRNA individually into the introgression strains. As expected, neither the *C. briggsae*-specific nor the *C. nigoni*-specific gRNA alone induced TR, i.e., 0 out of 166 screened progenies with the *C. briggsae-*specific gRNA and 0 out of 149 screened progenies with the *C. nigoni-*specific gRNA (Fig. 5B and Additional File 2: Table S2). The results confirmed that HR was somehow suppressed in the introgression strains. However, when we simultaneously injected both gRNAs into the gonads of the introgression line, a total of 12 targeted recombinants out of 190 screened progenies were obtained with a targeted recombinant frequency of approximately 6.31% (*p* < 0.01, Fisher’s exact tests with multiple testing correction using FDR; Fig. 5B and Additional File 2: Table S2). We randomly sequenced the recombination boundary of one of the targeted recombinants (ZZY10460). Again, we observed the deletion of 4–5 and 10–11 nucleotides in the expected target site from the *C. briggsae* and *C. nigoni* genomes, respectively, in the recombinant (Fig. 5C), supporting the idea that recombination was achieved through NHEJ. Taken together, the results demonstrated that efficient TR could be readily achieved in both hybrid F1 and introgression strains independently of sequence homology through the simultaneous injection of both *C. briggsae-* and *C. nigoni-*specific gRNAs.

## Discussion

In this study, we demonstrated the feasibility of leveraging the CRISPR/Cas9 system with dual gRNAs to achieve TR in hybrids between *C. briggsae* and *C. nigoni*. The method greatly facilitates the mapping of HI loci by circumventing the suppression of recombination between homologous chromosome arms, especially in the introgression line. We showed that a single gRNA was sufficient to induce TR in a region with relatively high sequence similarity in the F1 hybrids but not in the introgression strain (Fig. 2). The application of dual gRNAs respectively targeting *C. briggsae* and *C. nigoni* syntenic regions significantly increased the efficiency of TR in both the F1 and introgression strains, regardless of sequence homology and genetic background, supporting the idea that the dual gRNAs induced recombination through NHEJ (Figs. 3, 4, and 5). This method paves the way for high-resolution genetic mapping of HI loci, not only between *C. briggsae* and *C. nigoni* but also between any species or populations in which significant sequence divergence and/or genome rearrangements are common.

Although we and others identified numerous HI phenotypes in hybrids between the nematodes *C. briggsae* and *C. nigoni* [8, 25, 26], our attempts to identify HI genes between *C. briggsae* and *C. nigoni* have made little progress over the past decade, largely due to our inability to further narrow down the introgression intervals through crossing [8, 25]. Unlike HR-based TR, which has been adopted for genetic mapping in a variety of intraspecies in which sequence divergence is minimal [15, 16, 22, 23], there are substantial sequence divergence and wide-spread genomic rearrangement events between *C. briggsae* and *C. nigoni* that are likely to present significant barriers to HR. We showed that CRISPR/Cas9 with a single species-specific gRNA induced TR in the hybrid F1 but not in the introgression strains (Figs. 2B and 5B). The failure of TR in the introgression strain was unlikely to be caused by the gRNA used because the same gRNA and identical target sites were used in both the hybrid F1 and the introgression strains. Rather, it could have occurred because of synapsis defects that prevented crossover events. Crossover depends on proper formation of synapsis followed by homologous pairing, which are initiated at the pairing center. The centers are located at the end of different chromosomes on different arms, which are bound by multiple DNA-binding proteins to initiate synapsis in *C. elegans* [27]. The DNA-binding proteins involved in synapsis are fast evolving and are derived from recent duplications in *C. elegans*. It is well established that there is a marked divergence in the regulation of synapsis and crossing-over between nematodes [28]. It is possible that these species-specific DNA-binding proteins may not be functionally compatible between *C. briggsae* and *C. nigoni*. On top of this, the sequences of the pairing centers are likely to be divergent from each other. Therefore, the regulatory control of synapsis is likely to be relaxed due to incompatibilities in either of the DNA-binding proteins or sequences of the pairing centers or their combination, leading to the observed TR with a single gRNA in the F1 hybrids. However, given that the introgression strain is essentially a *C. nigoni* strain except carrying a genomic fragment from *C. briggsae*, it is possible that the DNA-binding proteins required for the initiation of synapsis are largely intact, providing robust control over crossover events by regulating the formation of pairing centers, for example, by demanding a high sequence homology to initiate synapsis or stabilize the homolog pair. Consistent with this, *him-8*, as one of the genes encoding the DNA-binding proteins, was located at the pairing center and indispensable for X chromosome disjunction [29]. Interestingly, *Cbr-him-8* was also reported to be a hybrid incompatibility gene leading to F1 male-specific lethality in the hybrids between *C. briggsae* and *C. nigoni* although the result remains controversial [30, 31].

On the contrary, CRISPR/Cas9 coupled with dual gRNAs facilitates TR by inducing discrete DSBs on two parental chromosomes in both F1 hybrids and introgression strains. The DSBs trigger TR through inter-chromosomal NHEJ between homologous chromosomes in a manner independent of sequence homology. Dual gRNA treatments were also employed to generate deletions of various sizes within species [32–34]. A portion of these deletions are expected to be derived from the fusion of homologous chromosomes. If the two gRNAs target different parts of homologous chromosomes, the fusion of the homologous chromosomes is expected to generate a deletion in one chromosome and an insertion in the other. Notably, HR-based recombination only works well when the gRNA-targeting site is disrupted after recombination. Otherwise, the recombinant chromosome is still subject to subsequent cutting, leading to a reduced frequency of recombination events. Therefore, the observed HR with dual gRNAs may only account for a small proportion of TR when the gRNA cannot differentiate between two homologous chromosomes.

Another attractive way of increasing HR frequency is to knock out crossover-suppression genes, such as *RECQ4* and FIGL1 [35]. Simultaneous mutations in the two genes significantly improve crossover efficiency in plants [35]. It would be interesting to explore whether coupling crossover-suppression-gene knockdown with dual gRNAs can further increase TR efficacy in the future. Dual gRNAs were also reported for the creation of structural variations such as duplications or translocations [36, 37]. Translocation mainly results from NHEJ or alterative end-joining (a-EJ) that requires no or minimal homology [38]. Previous studies have shown that the knockdown of components required for NHEJ elevated the translocation frequency, partially due to the increased efficacy of a-EJ [39]. It would be thus interesting to test whether the depletion of such factors can further increase the efficiency of TR induced by dual gRNAs.

One caveat of dual gRNA-mediated TR is that the fusion of the broken DNA ends is prone to errors and commonly produces unintended mutations, as reported previously [18, 32, 34]. DSBs induced by CRISPR/Cas9 system often result in variations encompassing large genomic regions. For example, we frequently observed large deletions in our recombinants (Figs. S2 and S3). One of the possible reasons is that the Cas9 protein can remain bound to the broken DNA ends, potentially affecting the repair of the broken chromosomes [40]. Therefore, genomic sequencing of the recombinants is highly recommended for subsequent functional analysis.

## Conclusions

In summary, TR with dual gRNAs enables researchers to achieve high-resolution genetic mapping that is independent of sequence homology regardless of genetic background within or between species. This is especially relevant to the mapping of HI factors between species, which are commonly located within highly divergent genomic regions, such as species-specific repetitive sequences [41]. The method is expected to be applicable to any other situations that demand fine genetic mapping and when recombination is suppressed because of a unique genetic background, a lack of sequence homology or the presence of a genomic rearrangement within or between species.

## Methods

### Worm maintenance and strains

All worm strains were maintained under 25 °C on plates with nematode growth medium except with double concentration of agar, which were pre-seeded with *Escherichia coli* OP50. The introgression lines were generated by repeatedly backcrossing individual GFP marker (*Cbr-pmyo-2::gfp*) randomly inserted into the *C. briggsae* (AF16) genome to the genome of wild-type *C. nigoni* (JU1421) as described [8, 25]. Details on the introgression location and size for the 112 lines were listed in Additional File 2: Table S1. For generation of TR on chromosome IV in hybrid F1 and introgression strain, the transgenic *C. briggsae* carrying a GFP marker (ZZY0734) was used, which was generated by an optimized *miniMos* transgenesis method [42]. The GFP insertion site was on the right arm of the *C. briggsae* chromosome IV. For the generation of TR on chromosome II in hybrid F1, the transgenic *C. briggsae* carrying a mCherry marker inserted at the right arm of chromosome II (ZZY0782) was used. For the generation of TR in hybrid F1 animals, 10 transgenic *C. briggsae* males were mated with 10 wild-type *C. nigoni* females to produce hybrid F1 animals. Those young-adult female progenies expressing the GFP marker were further backcrossed to *C. nigoni* for at least 5 generations to remove any marker unlinked *C. briggsae* genomes for genotyping with swPCR or genome sequencing. For the generation of TR in introgression strains, a *C. nigoni* strain, ZZY10458, which carries a large GFP-linked genomic fragment from the *C. briggsae* chromosome IV, was first generated by repeatedly backcrossing ZZY0734 to *C. nigoni*. The introgression size was estimated to be roughly 10 Mb on the right arm of *C. briggsae* chromosome IV as determined by swPCR. CRISPR/Cas9 RNPs were microinjected into the gonads of ZZY10458 young adult female as detailed below.

### CRISPR/Cas9 and gRNAs injection

CRISPR/Cas9 proteins, trans-activation RNA (transRNA), and CRISPR RNA (crRNA) were all purchased from Integrated DNA Technologies (Coralville, IA, USA). The sequences of all gRNAs used in this study were listed in Additional File 2: Table S3 [43–45]. To generate CRISPR/Cas9 RNP, 2 μl of 100 μM transRNAs was mixed with 2 μl of 100 μM crRNAs followed by incubation at 90 °C for 5 min. The mixture was cooled to room temperature to generate the annealed gRNA duplexes. Cas9 proteins (2 μl, 10 μg/μl) was mixed with gRNA duplexes to generate the final RNP complexes, which was microinjected into distal ends of both gonads of young adult females by microinjection [46]. For each targeting gene/site, 3 technical replicated injections were performed (Additional File 2: Table S2). Technical replicates were defined as three sessions of injection performed with the injection mixtures freshly made with identical recipe each time and by the same person. Successfully injected females were mated with wild-type *C. nigoni* males. The marker-expressing progeny were screened for the presence of expected recombination by swPCR (Fig. 2A). As a control, we also screened marker-expressing progeny derived from the similar crossings except that the gRNAs were not included in microinjection in three replicates. To validate whether the results from each replicate are reproducible between one another, Fisher’s exact test with multiple testing correction was performed for all the results between replicates. No significant difference was observed between replicates (Additional File 1: Fig. S5). For simplicity reason, only the results of accumulative TR frequency between hybrid F1 and introgressions were shown in the main figure. The same strategy was applied to all the comparisons of TR frequency at other target sites.

### Screen for targeted recombinants

To screen for targeted recombinants, we amplified two *C. briggsae*-specific genomic fragments by swPCR, one being upstream and the other downstream of the expected gRNA targeting site (Fig. 2A). Both amplified fragments were within 2 kb away from the targeting site. We only screened for the GFP- or mCherry expressing recombinants, whose upstream and downstream parts of the recombined site would be *C. nigoni* and *C. briggsae* genome respectively. Therefore, swPCR results were expected to be negative and positive for the upstream and downstream fragments respectively. The targeted recombinants were further verified by PCR amplification of at least 5 more regions both upstream and downstream of the gRNA target sites. The full list of the species-specific swPCR primers used in this study was shown in Additional File 4: Table S4. The TR efficiency was calculated by dividing the number of confirmed targeted recombinants by the number of total worms genotyped with swPCR from all the three injections. TR efficiencies for all the injections were listed in Additional File 2: Table S2.

### Sequencing of recombination boundaries

The precise recombination boundaries were determined either by Sanger sequencing or the genomic DNA sequencing reads from Oxford Nanopore sequencing. For Sanger sequencing, genomic regions flanking the expected recombination boundary were PCR amplified using two primers: one is specific to the *C. briggsae* genome and the other specific to the *C. nigoni* genome. To test the specificity of the primers, we performed PCR with the primers using either *C. briggsae* or *C. nigoni* genomic DNAs, and we observed no band, indicating the primers were specific to the TR region. Nested primers were used to increase the specificity of amplification if multiple bands were associated with the initial PCR. The primers used for amplifying and sequencing of recombination boundaries were listed in Additional File 3: Table S5. Amplified target fragments were gel purified with FastPure Gel DNA extraction mini kit (Vazyme) before Sanger sequencing.

For Oxford Nanopore sequencing, high molecular weight (HMW) genomic DNAs were extracted using Gentra Puregene Cell Kit (QIAGEN). The genomic DNAs were sequenced on an Oxford Nanopore MinION device (Rev D, FLO-MIN106) using the standard genomic DNA sequencing by ligation method (SQK-LSK109). Raw sequencing signal files (FAST5) were basecalled using Guppy (v5.0.7) (https://nanoporetech.com/) with high-accuracy model. “–qscore_filtering” option was included for categorizing reads as “pass” or “fail” with default cutoff. Only passed reads were retained for further analysis. The filtered reads were mapped against the *C. briggsae* genome (CB5) and *C. nigoni* genome (CN3) using minimap2 (v2.17) [47] with default parameters. The *C. briggsae* reads coverage and the recombination boundary-spanning reads were manually checked with Integrative Genomics Viewer (IGV) (v2.4.16) [48]. Boundary-spanning reads were identified by manually selecting the *C. briggsae* boundary reads with a soft clipped sequence but was able to be aligned to the *C. nigoni* syntenic region. Boundary-spanning reads were further extracted using SAMtools (v1.9) [49] and mapped to manually constructed recombination junction templates for visualization. Read statistics was analyzed using SeqKit (v0.13.2) [50]. The sequencing reads statistics were listed in Additional File 3: Table S6.

### One-to-one ortholog identification and sequence extraction

The orthologous genes between *C. briggsae* and *C. nigoni* were identified by Othorfinder (v2.15) [51]. The one-to-one orthologs were extracted similarly as described [41]. The intergenic regions are defined as the combination of the upstream and downstream sequences of a specific orthologous gene. The upstream sequence of an orthologous gene is defined as the genomic interval between the start codon of the gene of interest and the start or stop codon of its immediately upstream neighboring gene, depending on its orientation. Similarly, the downstream sequence of an orthologous gene is defined as the genomic interval between the stop codon of a gene of interest and the start or stop codon of its immediately downstream neighboring gene, depending on its orientation. Sequence similarities were calculated by BLASTn (v2.11.0) [52] with “-max_target_seqs 1” option. To account for similarity over the entire region, the output was weighted by sequence length. Specifically, the score was multiplied by the ratio between the alignable length and the length of the larger input out of the orthologous pair. An orthologous pair was considered as unalignable if there was no output from the BLASTn alignment. The sequence extraction, alignment, and the calculation of alignment-length-weighted similarity score of the CDS, intron, and intergenic region of each ortholog pair were performed with customized python scripts, which were deposited on GitHub (https://github.com/JeffreyXIE/ortholog_seq_extract/).

### Statistical analyses

Wilcoxon ranked sum test was performed for the comparison of introgression sizes and sequence similarities. Chi-square test or Fisher’s exact test were performed for the comparison of targeted recombinant frequency. Multiple comparisons were corrected using the false discovery rate (FDR) method. The 95% confidence interval of targeted recombinant frequency is calculated using the Agresti-Coull method.

### Supplementary Information

**Additional file 1: Figure S1.** Targeted recombinant frequency is significantly higher for the genes with elevated sequence homology regardless of its genomic position. Top: bar plot showing the comparison of targeted recombinant frequency between genes with relatively high and low homology. Bottom: four genes with relatively high sequence homology, including *Cbr-fan-1*, *CBG05865*, *Cbr-nep-16* and *Cbr-gtl-1*, are located on the middle of the chromosome IV, whereas one gene with relatively low homology is located on the right arm of chromosome IV. Error bar represents 95% confidence interval calculated as in Fig. 2B (****) *p* < 0.001; (***) *p* < 0.001 (Fisher’s exact test with multiple testing correction using the FDR method). **Figure S2.** Targeted recombination in the proximity of an inversion induced by dual gRNAs. (A) The regions flanking the dual gRNAs target sites are shown as in Fig. 4. Note that recombination was also achieved between *C. briggsae* gene *CBG16437* (around 4 kb downstream of the *C. briggsae-*specific gRNA targeting site) and *C. nigoni* gene *g17641* (around 90 kb from the *C. nigoni* gRNA targeting sites). The recombination boundaries are highlighted with dashed parallelogram. (B) Confirmation of the recombination through Oxford Nanopore sequencing. Only the sequencing reads that span the recombination boundaries are shown. **Figure S3.** Targeted recombination induced by dual gRNAs associated with a large deletion. (A) Shown is an inverted alignable ortholog pair with gRNA target sites in *C. briggsae* (*CBG16450*) and *C. nigoni* (*g17645*) indicated. (B) Nanopore sequencing reads reveal a deletion of *C. briggsae* sequence around 90 kb in size in a recombinant induced by the dual gRNAs. **Figure S4.** The Integrative Genomics Viewer (IGV) track view of the recombination boundary reads (Oxford Nanopore sequencing) in the targeted recombinants. The parts of manually constructed recombination boundary that belong to the genome of *C. nigoni and C. briggsae* are highlighted in orange and blue, respectively. The names of the boundary-spanning reads are also indicated. **Figure S5.** Targeted recombinant frequencies are reproducible across the three replicates for each gRNA target. Shown are the bar plots of targeted recombinant frequency for the three replicates of each gRNA target as listed in Additional File 2: Table S2. The total number of screened worms (n) and the *p* values are indicated. Note that no significant differences were observed between each pair of the three replicates for all the injections. (Fisher’s exact test with multiple testing correction using FDR method). Error bar represents 95% confidence interval calculated as in Fig. 2B.

**Additional file 2: Table S1.** List of introgression size for the 112 independent introgression lines that carry a GFP-linked C. briggsae chromosomal fragment in an otherwise C. nigoni background.

**Additional file 3: Table S2.** Targeted recombination statistics. **Table S3.** List of gRNA sequences with their targeting genes, derived recombinants, specificity score and predicted efficiency in this study. **Table S5.** List of primers for PCR and Sanger sequencing of the targeted recombination site. **Table S6.** Read statistics of Oxford nanopore sequencing of recombinant genomic DNAs.

**Additional file 4: Table S4.** List of species-specific primers used for single worm PCR in this study.

## Data Availability

All data generated or analyzed during this study are included in this published article, its supplementary information files, and publicly available repositories. The *C. briggsae* genome (CB5) and *C. nigoni* genome (CN3) assembled by our group with corresponding gene annotation files can be found on NCBI BioProject database (https://www.ncbi.nlm.nih.gov/bioproject) under accession number PRJNA917437 and GitHub (https://github.com/PikaPatch/CB-CN). The Oxford Nanopore sequencing raw reads generated in this study were submitted to the NCBI BioProject database under accession number PRJNA947947.

## Abbreviations

HR: Homology-based recombination
HI: Hybrid incompatibility
TR: Targeted recombination
gRNA: Guide RNA
transRNA: Transactivations RNA
crRNA: CRISPR RNA
DSB: DNA double-strand break
swPCR: Single-worm PCR
NHEJ: Non-homology-based end joining
a-EJ: Alterative end-joining
FDR: False Discovery Rate
