# Supplementary material for: Efficient targeted recombination with CRISPR/Cas9 in hybrids of Caenorhabditis nematodes with suppressed recombination
Source: BMC Biol. 2023 Sep 29;21:203. doi: 10.1186/s12915-023-01704-0 (PMC10542263; doi:10.1186/s12915-023-01704-0)
Supplement: Supplementary file 1 — Additional file 1: Figure S1. Targeted recombinant frequency is significantly higher for the genes with elevated sequence homology regardless of its genomic position. Top: bar plot showing the comparison of targeted recombinant frequency between genes with relatively high and low homology. Bottom: four genes with relatively high sequence homology, including Cbr-fan-1, CBG05865, Cbr-nep-16 and Cbr-gtl-1, are located on the middle of the chromosome IV, whereas one gene with relatively low homology is located on the right arm of chromosome IV. Error bar represents 95% confidence interval calculated as in Fig. 2B (****) p < 0.001; (***) p < 0.001 (Fisher’s exact test with multiple testing correction using the FDR method). Figure S2. Targeted recombination in the proximity of an inversion induced by dual gRNAs. (A) The regions flanking the dual gRNAs target sites are shown as in Fig. 4. Note that recombination was also achieved between C. briggsae gene CBG16437 (around 4 kb downstream of the C. briggsae-specific gRNA targeting site) and C. nigoni gene g17641 (around 90 kb from the C. nigoni gRNA targeting sites). The recombination boundaries are highlighted with dashed parallelogram. (B) Confirmation of the recombination through Oxford Nanopore sequencing. Only the sequencing reads that span the recombination boundaries are shown. Figure S3. Targeted recombination induced by dual gRNAs associated with a large deletion. (A) Shown is an inverted alignable ortholog pair with gRNA target sites in C. briggsae (CBG16450) and C. nigoni (g17645) indicated. (B) Nanopore sequencing reads reveal a deletion of C. briggsae sequence around 90 kb in size in a recombinant induced by the dual gRNAs. Figure S4. The Integrative Genomics Viewer (IGV) track view of the recombination boundary reads (Oxford Nanopore sequencing) in the targeted recombinants. The parts of manually constructed recombination boundary that belong to the genome of C. nigoni and C. briggsae are highlighted in orange [file 12915_2023_1704_MOESM1_ESM.pdf]

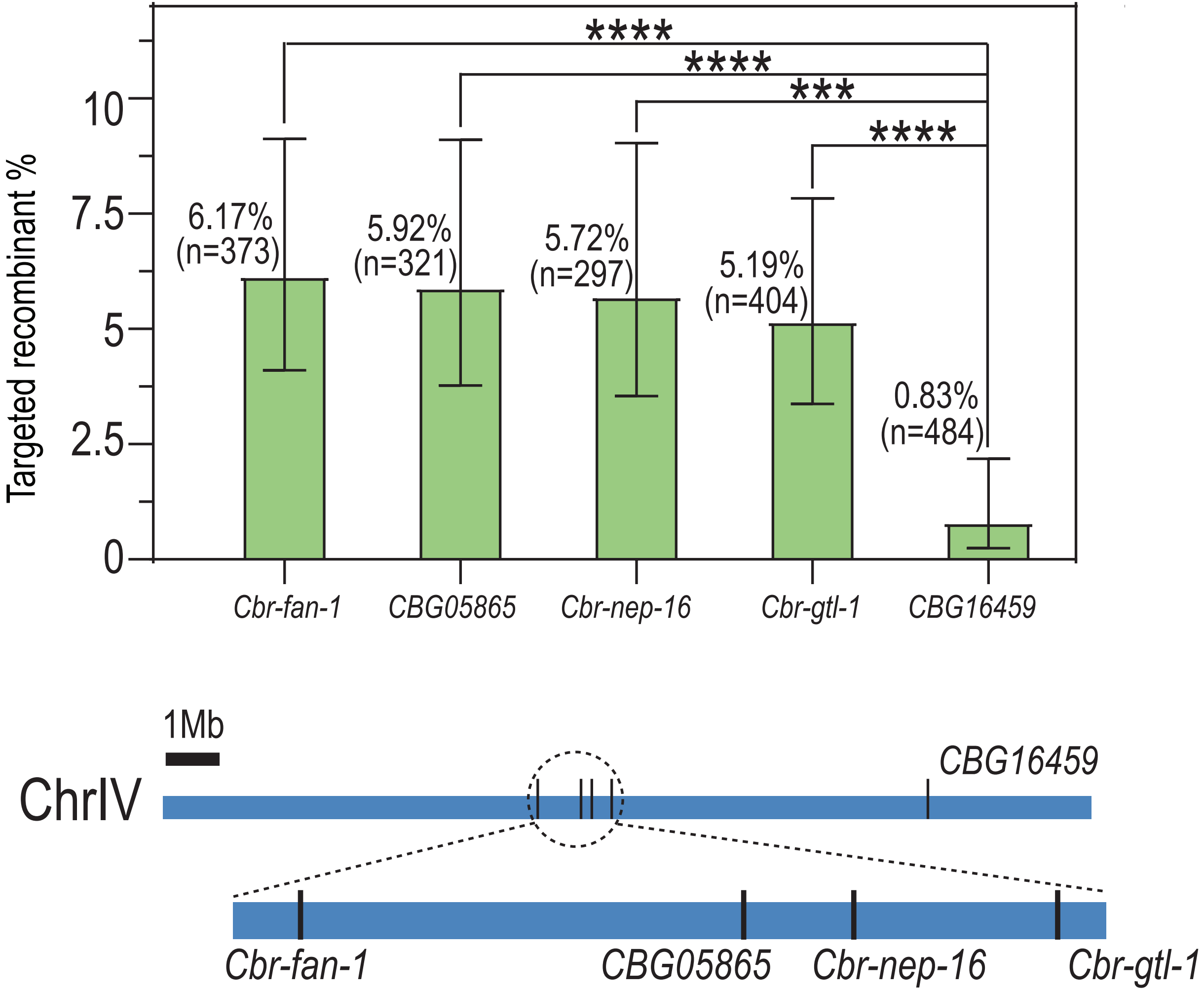

Targeted recombinant %
0
2.5
5
7.5
10
****
****
***
****
6.17%
(n=373)
5.92%
(n=321)
5.72%
(n=297)
5.19%
(n=404)
0.83%
(n=484)
Cbr-fan-1
CBG05865
Cbr-nep-16
Cbr-gtl-1
CBG16459
1Mb
ChrIV
CBG16459
Cbr-fan-1
CBG05865
Cbr-nep-16
Cbr-gtl-1

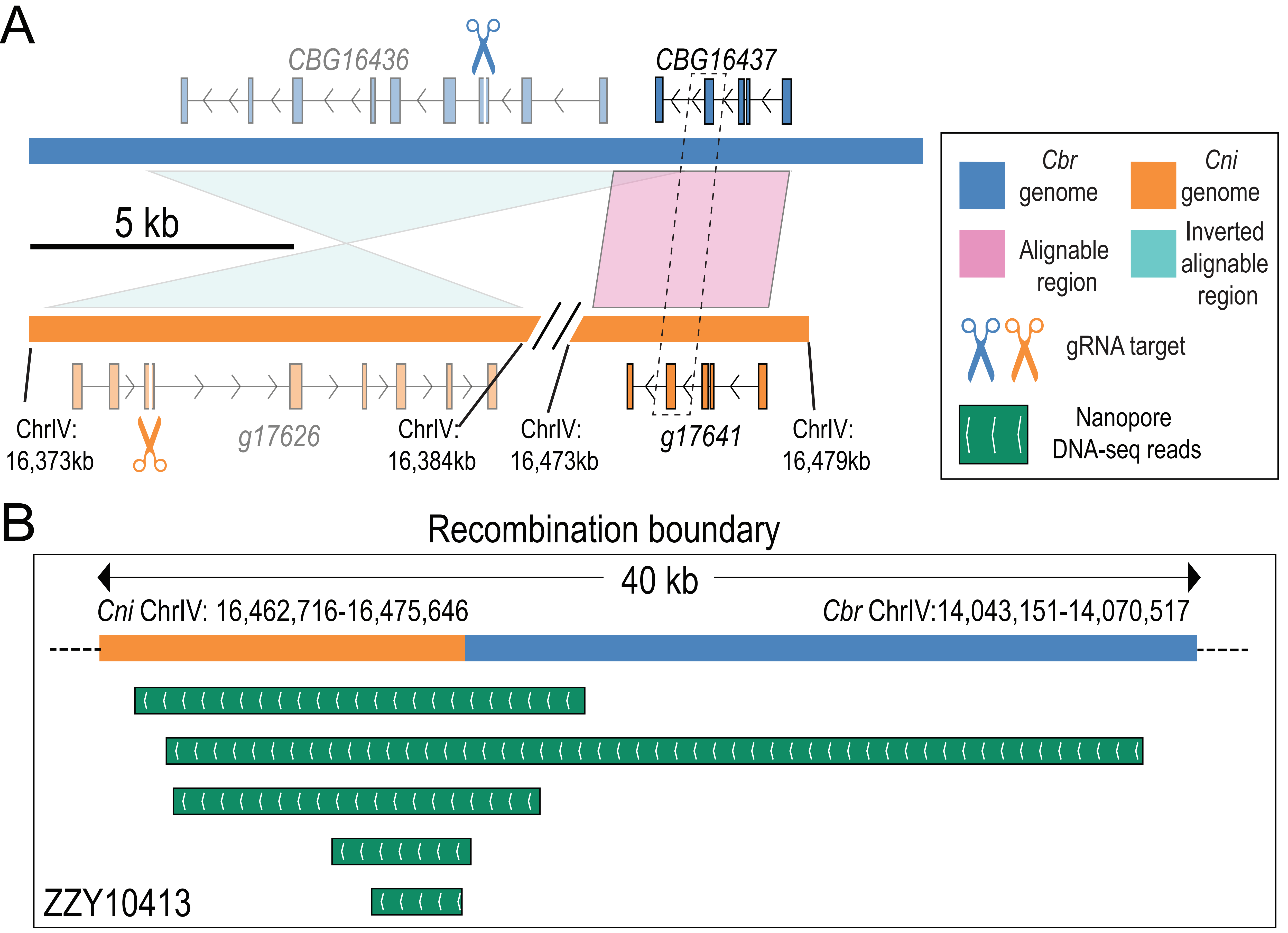
A
CBG16436
CBG16437
5 kb
ChrIV: 16,373kb
g17626
ChrIV: 16,384kb
ChrIV: 16,473kb
g17641
ChrIV: 16,479kb
Cbr genome
Cni genome
Alignable region
Inverted alignable region
gRNA target
Nanopore DNA-seq reads
B
Recombination boundary
40 kb
Cni ChrIV: 16,462,716-16,475,646
Cbr ChrIV:14,043,151-14,070,517
ZZY10413

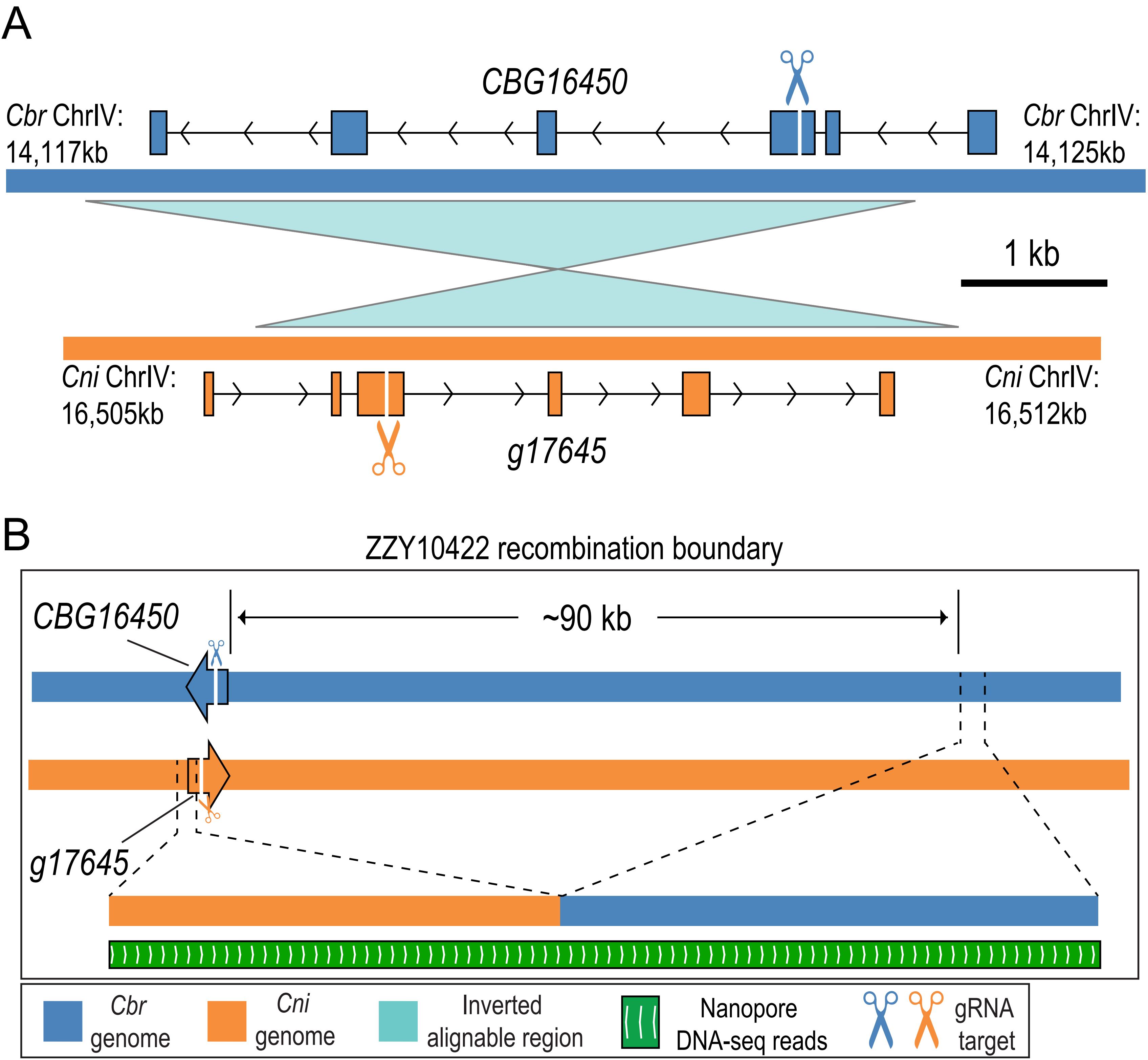
A
CBG16450
Cbr ChrIV: 14,117kb
Cbr ChrIV: 14,125kb
1 kb
Cni ChrIV: 16,505kb
Cni ChrIV: 16,512kb
g17645
B
ZZY10422 recombination boundary
CBG16450
~90 kb
g17645
Cbr genome
Cni genome
Inverted alignable region
Nanopore DNA-seq reads
gRNA target

ZZY10412 recombination boundary

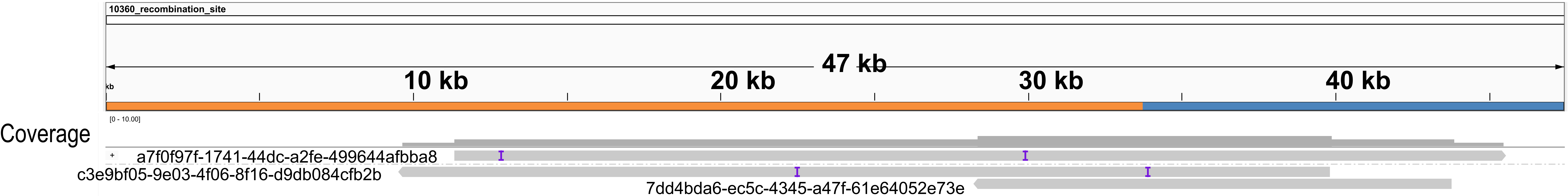

ZZY10413 recombination boundary

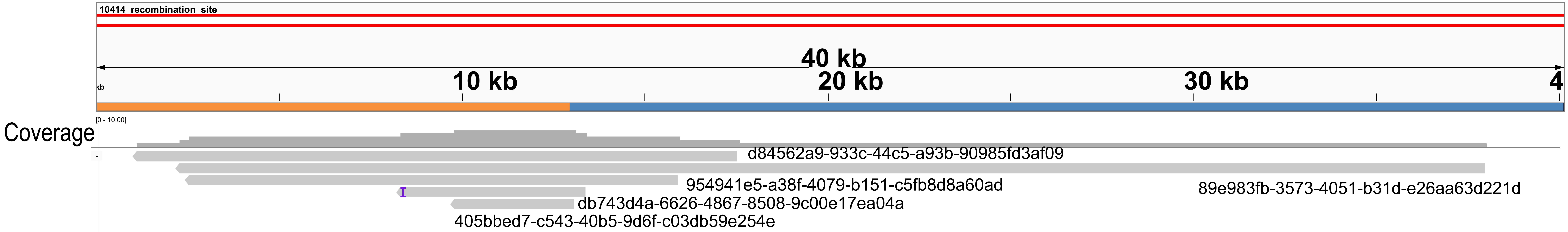

ZZY10422 recombination boundary

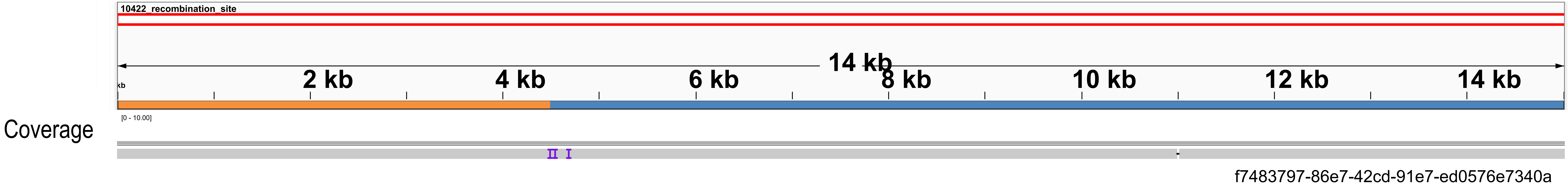

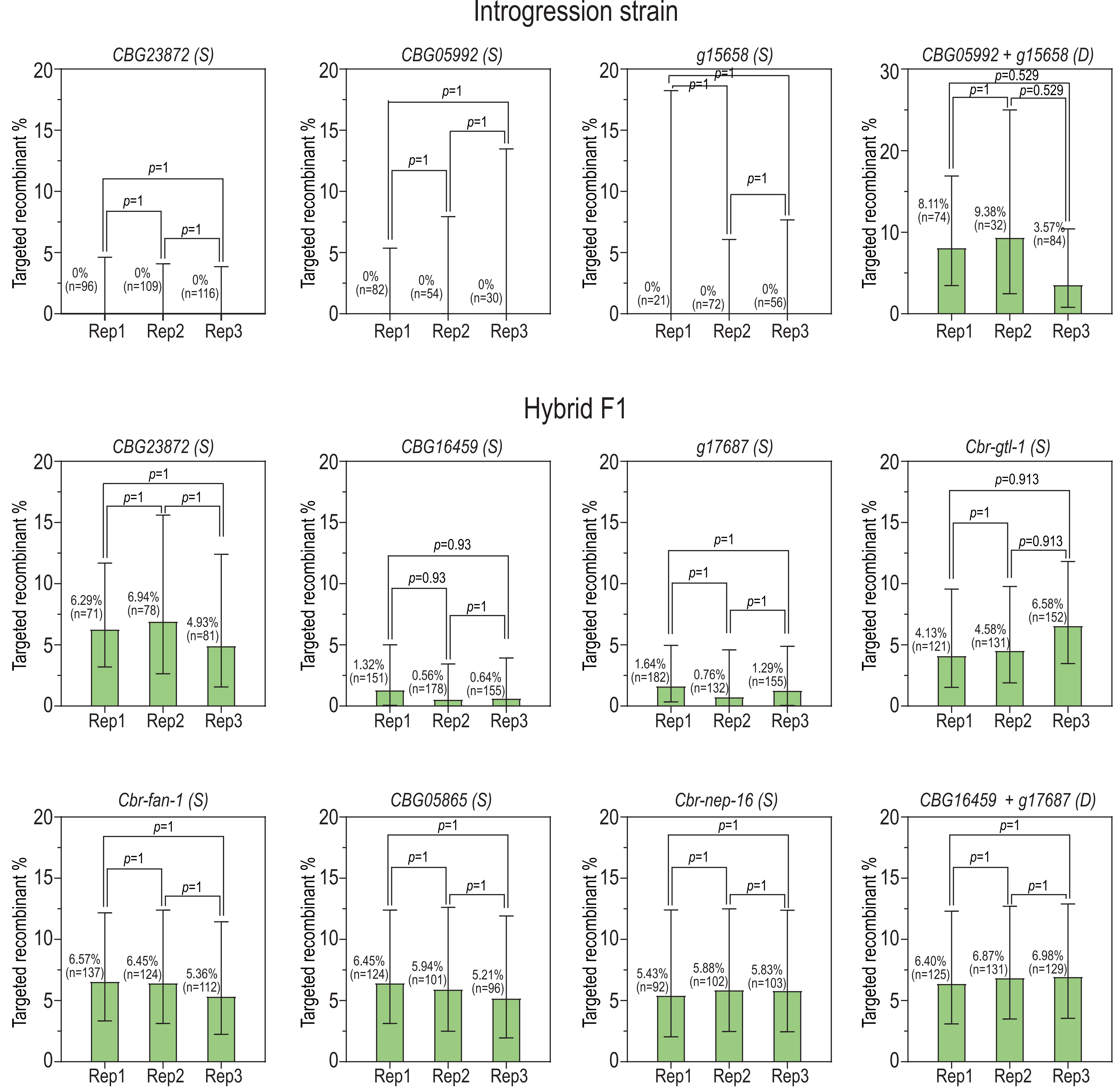
Introgression strain
CBG23872 (S)
0% (n=96)
0% (n=109)
0% (n=116)
CBG05992 (S)
0% (n=82)
0% (n=54)
0% (n=30)
g15658 (S)
0% (n=21)
0% (n=72)
0% (n=56)
CBG05992 + g15658 (D)
8.11% (n=74)
9.38% (n=32)
3.57% (n=84)
p=0.529
p=1
p=0.529
Hybrid F1
CBG23872 (S)
6.29% (n=71)
6.94% (n=78)
4.93% (n=81)
CBG16459 (S)
1.32% (n=151)
0.56% (n=178)
0.64% (n=155)
p=0.93
p=0.93
p=1
g17687 (S)
1.64% (n=182)
0.76% (n=132)
1.29% (n=155)
Cbr-gtl-1 (S)
4.13% (n=121)
4.58% (n=131)
6.58% (n=152)
p=0.913
p=1
p=0.913
Cbr-fan-1 (S)
6.57% (n=137)
6.45% (n=124)
5.36% (n=112)
CBG05865 (S)
6.45% (n=124)
5.94% (n=101)
5.21% (n=96)
Cbr-nep-16 (S)
5.43% (n=92)
5.88% (n=102)
5.83% (n=103)
CBG16459 + g17687 (D)
6.40% (n=125)
6.87% (n=131)
6.98% (n=129)
p=1
Targeted recombinant %
Rep1
Rep2
Rep3
