# Supplementary material for: Efficient targeted recombination with CRISPR/Cas9 in hybrids of Caenorhabditis nematodes with suppressed recombination
Source: BMC Biol. 2023 Sep 29;21:203. doi: 10.1186/s12915-023-01704-0 (PMC10542263; doi:10.1186/s12915-023-01704-0)
Supplement: Supplementary file 3 — Additional file 3: Table S2. Targeted recombination statistics. Table S3. List of gRNA sequences with their targeting genes, derived recombinants, specificity score and predicted efficiency in this study. Table S5. List of primers for PCR and Sanger sequencing of the targeted recombination site. Table S6. Read statistics of Oxford nanopore sequencing of recombinant genomic DNAs. [file 12915_2023_1704_MOESM3_ESM.docx]

**Table S2. Targeted recombination statistics.**

| Targeting site (genes) | | Targeted recombinant frequency |
| --- | --- | --- |
| Introgression strains |  |  |
| *CBG23872* (S)  *CBG05992* (S)  *g15658* (S)  *CBG05992+g15658* (D) | | 0% (321)*  0% (166)  0% (149)  6.31% (190) |
| F1 hybrids | |  |
| *CBG23872* (S) | | 6.08% (296) |
| *CBG16459* (S) | | 0.83% (484) |
| *g17687* (S)  *Cbr-gtl-1* (S)  *Cbr-fan-1* (S) | | 1.27% (469)  5.19% (405)  6.17% (373) |
| *CBG05865* (S)  *Cbr-nep-16* (S) | | 5.92% (321)  5.72% (297) |
| *CBG16459+ g17687* (D) | | 7.26% (385) |
|  | |  |
| *CBG16436+ g17626* (D) | | 10.2% (98) |
|  | |  |
| *Cbr-otub-3+ g17645* (D) | | 5.5% (127) |

* Number of animals screened. S: single gRNA treatment. D: dual gRNAs treatment.

**Table S3. List of gRNA sequences with their targeting genes, derived recombinants, specificity score and predicted efficiency in this study.**

| Type | gRNA Sequence 5’ to 3’ | Targeting gene | Species | Recombinant strains | MIT^a^ | CFD^b^ | Doench^c^ |
| --- | --- | --- | --- | --- | --- | --- | --- |
| Single gRNA | TTTGTAGAATTCAAAGGATC | *CBG23872* | *Cbr* | ZZY10396 ZZY10397 ZZY10403 | 96 | 97 | 46 |
| Single gRNA | ATGTGCCACTCTGTGTAGAA | *Cbr-gtl-1* | *Cbr* | ZZY10452  ZZY10453 | 100 | 99 | 49 |
| Single gRNA | CCTCATCCCGAAATACAAAT | *cbr-fan-1* | *Cbr* | ZZY10457  ZZY10458 | 99 | 99 | 50 |
| Single gRNA | TTCAAGATTCAACCACCAAT | *CBG05865* | *Cbr* | ZZY10487  ZZY10488  ZZY10489 | 99 | 100 | 48 |
| Single gRNA | AAACGAACATCATGTACATC | *Cbr-nep-16* | *Cbr* | ZZY10479 | 99 | 100 | 50 |
| Dual gRNAs | TTGCTCACGTTCGTAGAATC | *CBG16459* | *Cbr* | ZZY10406  ZZY10408  ZZY10409 | 99 | 100 | 52 |
|  | TTCTCAATGCTGGCGATGGT | *g17687* | *Cni* |  | 100 | 100 | 54 |
| Dual gRNAs | GCTCAAACAGAACGCCACGA | *CBG16436* | *Cbr* | ZZY10412  ZZY10413 | 99 | 100 | 69 |
|  | GAACGGGATTCTGGCTTTTG | *g17626* | *Cni* |  | 99 | 99 | 36 |
| Dual gRNAs | TTCTTCTTCTTCATGAACGA | *Cbr-otub-3* | *Cbr* | ZZY10422 | 98 | 98 | 46 |
|  | ACGATGGGCGGGGCTTCTAC | *g17645* | *Cni* |  | 100 | 100 | 33 |
| Dual gRNAs | CAATGATAGTGATGAATGGA | *CBG05992* | *Cbr* | ZZY10460 | 91 | 98 | 64 |
|  | CATTCCACTCATTCTGAAGA | *g15658* | *Cni* |  | 96 | 98 | 57 |

a: MIT specificity score (Hsu *et al.* 2013) [2]; b: CFD specificity score (Tycko *et al.* 2019) [3]; c: Doench predicted efficiency (Doench *et al.* 2016) [4]

|  | | | | | | |
| --- | --- | --- | --- | --- | --- | --- |
|  | **Primer pairs (5'-3')** | | **Nested primer pairs (5'-3')** | **Sequencing primer (5'-3')** | **Recombinant strains** | |
| Forward | AGGGCTCCAATAGAGCAAGG | GATGAAGTGCCCACTTTAAAGGC | | AGTTGCTATATCTCGGAAAC | | ZZY10396 ZZY10397 ZZY10403 |
| Reverse | GCTAGAGAGGGGCCATCTTG | GTTGCTATATCTCGGAAACTAAGTTAGCTATC | |  |  |  |
| Forward | TTTTACTCCCTCGAACCGGC | CTAATATGCACCTAATGGGTTTTCTAGGCC | | CCACCAACTATTATTGCCTAG | | ZZY10406 ZZY10408 ZZY10409 |
| Reverse | TCACCCTTAAATCTTGTGCCT | CTGGAATGCAGGCATCGCTGAGAGTTTAC | |  |  |  |
| Forward | TCGCCAAGACTCTCGTTCAC | NA | | TCGCCAAGACTCTCGTTCAC | | ZZY10460  ZZY10461  ZZY10462 |
| Reverse | TCGTTCTTTCCATTCGGCCA |  |  | TCGTTCTTTCCATTCGGCCA | |  |
| Forward | CGGAGGACTCAAACCGAACA |  |  | CGGAGGACTCAAACCGAACA | |  |
| Reverse | CGACTAGTCCGACCATCAGC |  |  | CGACTAGTCCGACCATCAGC | |  |

**Table S5. List of primers for PCR and Sanger sequencing of the targeted recombination site.**

NA: not applicable

**Table S6. Read statistics of Oxford nanopore sequencing of recombinant genomic DNAs**

| **Strain** | **Total**  **number of**  **reads** | **Total**  **bases**  **(Gbp)** | **Mean read**  **length**  **(bp)** | **N50**  **length**  **(bp)** | **Max read**  **length (bp)** |
| --- | --- | --- | --- | --- | --- |
| ZZY10412 | 475,984 | 2.4 | 5,137.9 | 15,970 | 187,763 |
| ZZY10413 | 780,461 | 2.7 | 3,499.4 | 11,136 | 172,881 |
| ZZY10422 | 4,522,751 | 6.9 | 1,524.2 | 3,573 | 114,759 |
